# Acupuncture for Primary Dysmenorrhea: A Potential Mechanism from an Anti-Inflammatory Perspective

**DOI:** 10.1155/2021/1907009

**Published:** 2021-12-03

**Authors:** Wen-Yan Yu, Liang-Xiao Ma, Zhou Zhang, Jie-Dan Mu, Tian-Yi Sun, Yuan Tian, Xu Qian, Yi-Dan Zhang

**Affiliations:** ^1^School of Acupuncture, Moxibustion and Tuina, Beijing University of Chinese Medicine, Beijing 100029, China; ^2^The Key Unit of State Administration of Traditional Chinese Medicine, Evaluation of Characteristic Acupuncture Therapy, Beijing 100029, China

## Abstract

The low adverse effects of acupuncture for primary dysmenorrhea (PD), known as one of the most commonly reported gynecological debilitating conditions affecting women's overall health, have been thus far confirmed. Moreover, it has been increasingly recognized that inflammation is involved in such menstrual cramps, and recent studies have further shown that the anti-inflammatory effects of acupuncture are helpful in its control. This review portrays the role of inflammation in PD pathophysiology, provides evidence from clinical and animal studies on acupuncture for inflammation-induced visceral pain, and reflects on acupuncture-related therapies for dysmenorrhea with regard to their anti-inflammatory characteristics. Further research accordingly needs to be carried out to clarify the effects of acupuncture on proinflammatory factors in PD, particularly chemokines and leukocytes. Future studies on this condition from an anti-inflammatory perspective should be also performed in line with the notion of emphasizing stimulation modes to optimize the clinical modalities of acupuncture. Additionally, the effects and mechanism of more convenient self-healing approaches such as TENS/TEAS for PD should be investigated.

## 1. Introduction

Primary dysmenorrhea (PD), also called functional painful periods or menstrual cramps, is manifested as lower abdominal pains during menstruation without pelvic pathologies, accompanied by symptoms such as lumbago and leg pain, diarrhea, nervousness, fatigue, loss of appetite, and nausea and vomiting. PD usually occurs in adolescence after the menarche, and the pain starts a few hours before or immediately after menstruation and typically lasts for 8–72 hours [1, 2]. According to the latest epidemiological survey, about 45–90% of women in the world suffer from this condition during their menstrual period, among which 10–25% are of severe types [3]. Therefore, PD seems to be one of the most common gynecological disorders regardless of nationality and age [4]. About 1/3–1/2 of women with this debilitating disorder are also absent from work or school at least once per cycle, which significantly affects the quality of their work, study, and daily living activities, and even brings huge economic losses to any society [5, 6]. In addition, the occurrence of PD augments the risk of chronic pelvic inflammatory disease [4]. Although nonsteroidal anti-inflammatory drugs (NSAIDs) are currently the first-line medication for PD with well-grounded efficacy, the ineffective rate can reach about 20–30% [7]. Therefore, there is an urgent clinical demand to find a low-risk and effective nonpharmacological treatment option to relieve menstrual cramps.

Acupuncture has been popularly applied for a variety of diseases in China and many other countries as well, particularly for pain management [8, 9]. In fact, under the term “acupuncture”, there is a family of treatment modalities, including manual acupuncture, electroacupuncture (EA), moxibustion, acupoint catgut embedding (ACE) treatment, transcutaneous electrical acupoint stimulation (TEAS), transcutaneous electrical nerve stimulation (TENS) [10]. A large number of clinical trials and reviews have further provided evidence in support of acupuncture and its related therapies for PD [2, 11–15].

This alternative medicine has also demonstrated promising anti-inflammatory effects; therefore, it has been applied to treat inflammatory diseases [16–18], especially inflammatory pain [19–22]. Numerous studies have so far elucidated the underlying mechanism of acupuncture for various types of pain, including visceral pain [22, 23], and it has been emphasized that anti-inflammatory effects can contribute to the analgesic mechanism of this alternative therapy.

As a commonly seen among gynecological disorders, PD refers to a type of visceral pain, which develops closely related to inflammatory and immune factors [24–26]. An increasing number of studies have further examined the mechanism of acupuncture for PD from an anti-inflammatory perspective. This article aims to review the role of inflammation in PD pathophysiology and explore the potential anti-inflammatory analgesic mechanism of acupuncture as a form of alternative medicine to promote further research and optimize its clinical modalities.

## 2. Role of Inflammation in PD Pathophysiology

The menstrual cycle is manifested as a cyclical pattern of hormonal changes modulated by a feedback mechanism on the hypothalamus-pituitary-ovary (HPO) axis [27]. In the late secretory phase of the menstrual cycle, atrophic luteum accompanied by a rapid decline in hormone levels is the main regulatory factor shaping the destruction of the menstrual cascade [28]. Progesterone withdrawal also leads to the release of acid phosphatase and lysozyme from lysosomes into the cytoplasm. Arachidonic acid (AA) is further metabolized to prostaglandins (PGs) and leukotrienes (LTs) via the cyclooxygenase (COX) and lipoxygenase (LOX) pathways, respectively, both contributing to excessive myometrial contractions, giving rise to ischemia and hypoxia in the uterine muscle tissues [29]. The etiology of PD is multifaceted. Although numerous studies have been so far done in this respect, the pathophysiology of this alternative therapy has not been still fully clarified. The most generally acknowledged explanation is the concentration of PGs during menstruation [30]. In addition, a range of events including age, smoking habits, age at menarche, body mass index (BMI), alcohol abuse, family history, exercise, and the like can be among the risk factors for PD [4].

Notably, it has been suggested that menstruation is an inflammatory process [24, 25], before it, the endometrium exhibits inflammatory features of red with blood and edematous tissues, as a phenomenon associated with an influx of proinflammatory cytokines (viz. Interleukin-1 [IL-1], IL-6, and tumor necrosis factor-alpha [TNF*α*]) and leukocytes [28]. The inflammatory mediators are also an important part of the menstrual process [26], driven by a decrease in the levels of nonsteroid hormones in the late secretory phase of a nonconceptual cycle [28]. Based on previous studies, the relationship between inflammation and PD has recently attracted increasing attention even though people have a deeper understanding of the occurrence and development of PD. In the following, the relationship between PD pathophysiology and inflammation is being discussed.

### 2.1. Progesterone Withdrawal-Initiated Inflammation in Menstrual Cycle in PD

The current inflammatory perspective of the menstrual cycle in PD involves a complex set of events, driven by the fall in progesterone levels, which activate the release of nuclear factor-*κ*B (NF-*κ*B) from its inhibition by the inhibitor of I*κ*B, leading to the downstream transduction and translation of inflammatory genes and a release of proinflammatory mediators (that is, inflammatory cytokines, chemokines, and PGs) together with an influx of inflammatory cells [31–33]. Beyond that, a cascade of inflammatory events reflected in the activation of matrix metalloproteinases (MMPs) and other degradation enzymes can participate in maintaining inflammation and eventually cause tissue destruction.

Of note, progesterone plays an anti-inflammatory role in the menstrual cycle [34, 35]. The continued presence of progesterone can significantly inhibit excessive inflammatory events in the endometrium [36–38]. Distinct uterine inflammation manifested by substantial leukocyte content has been also shown in mice lacking progesterone receptors [36]. Progesterone additionally inhibits the production and activation of MMPs [39] and reduces the inflammatory responses induced by T-cell activation [35]. The reduction of ovarian hormone levels in the endometrium is typically associated with PD [40]. One other study have further revealed that imbalances in estradiol and progesterone could also affect the synthesis of PGF2*α* in the endometrium and lead to menstrual pain [41]. Therefore, progesterone may enhance PD prognosis by regulating the levels of cytokines, inhibiting the activation and migration of immune cells, and reducing the oxidant activity. The inflammatory response caused by progesterone withdrawal in the first phase of the menstrual cycle is thus involved in PD occurrence.

### 2.2. PGs-Mediated Inflammatory Response in PD

PGs are associated with inflammatory effects and they are considered as the fundamental mechanism for PD formation [30]. PGF2*α* can further cause vasoconstriction and result in a decrease in the blood flow, which in turn stimulate abnormal spastic contraction of uterine smooth muscles, and ultimately induce tissue ischemia and hypoxia, and pain [42]. The function of PGE_2_ depends on the type of receptors [43]. PGE_2_ mediated by the PGE_2_ receptor 2 also plays the role of relaxing vessels and inhibiting the contraction of uterine smooth muscles and may even work to increase edema and recruit leukocytes [44]. Studies in this line have reported that patients with PD experience significantly higher levels of PGF2*α* and PGE2 in the endometrial and menstrual blood than those without this condition [30]. There is even evidence that PGF2*α* can augment the sensitivity of nerve endings to pain and lower the pain perception threshold [45].

In addition, some studies have demonstrated that PGs can enhance the migration of inflammatory factors to the endometrium [44, 46]. PGF_2*α*_ can further promote neutrophils (NEUT) migration by increasing the release of CXCL1 [47], while PGE_2_ enhances leukocyte migration by inducing the expression of CXCL8 via NF-*κ*B signaling pathway [48, 49]. Additionally, the overexpression of inflammatory factors (such as TNF*α*, IL-1, and IL-6) can elevate the synthesis or release of PGF_2*α*_ in the uterus to trigger PD [50].

### 2.3. LTs and Inflammation in PD

LTs, as important inflammatory mediators, play an essential role in the PD process [51, 52]. Studies have accordingly shown that 10–30% of patients with PD have no obvious changes in PG levels in the uterus, while the content of LTs in the uterus and menstrual blood had significantly increased, and the LTE4 content in the urine of some young PD patients on the first day of menstruation was equivalent to three times that of women without this condition [53, 54]. LTs can also participate in the chemotaxis and activation of leukocytes, causing leukocytes to accumulate in the inflammatory area and release inflammatory mediators, which can lead to smooth muscle contractions and increased vascular permeability. Studies have further revealed that the use of LT antagonists can effectively relieve pain in patients with PD, whose traditional treatment with PG synthase inhibitors might not be much effective [55].

### 2.4. Changes of Cytokines and Chemokines in PD

The overexpression of cytokines and chemokines during the menstrual cycle drives the inflammatory microenvironment of PD in the uterus and plays a leading role in leukocyte recruitment.

#### 2.4.1. TNF*α*

TNF*α* is a potent proinflammatory cytokine that mediates complex biological responses, including the upregulation of inflammatory ones [50]. The role of TNF*α* in PD pathogenesis has been emphasized as stimulating the synthesis or release of PGs [50, 56], resulting in hypercontraction of the myometrium, which leads to ischemic pain. Studies have further reported that women with PD have higher plasma IL-6 and TNF*α* levels than healthy cases [57, 58]. Targeting TNF*α* and other factors to regulate arachidonic acid and inflammatory signaling pathways is thus assumed as an effective approach for PD treatment [59]. Moreover, the genotype of TNF*α*-308 GG may be a useful tool for predicting PD susceptibility [60].

#### 2.4.2. IL

Plasma cytokine levels, including IL-1*β*, IL-6, and IL-10, have been significantly altered in women with a normal menstrual cycle [61]. IL-1*β* and IL-6 levels have been also negatively relevant to estradiol and progesterone levels, indicating the involvement of immune inflammation in the menstrual cycle [62]. Studies have additionally shown that the IL-6 level in the luteal phase was significantly higher than that in follicular one [63], and plasma IL-6 concentration significantly increased in patients with PD on the first day of menstruation, resulting in enhanced uterine muscle contractions and reduced uterine blood flow [64, 65]. Research has further found that a reduction in IL-6 levels and an increase in anti-inflammatory factors can be induced by aerobic exercises, therefore relieving PD [50].

#### 2.4.3. Eotaxin

Eotaxin, also known as C–C motif chemokine ligand 11 (CCL11), is a member of the CC subfamily of chemokines and acts after binding to C–C motif chemokine receptor 3 (CCR3). As a specific chemokine of eosinophil (EOS), eotaxin has the strongest chemotactic activity [66] and it has been confirmed that the local injection of CCL11 can significantly augment EOS in local tissues [67]. A recent study has shown that patients with PD have significantly higher eotaxin levels in their blood than healthy individuals, suggesting that eotaxin may be involved in PD development [68].

During the menstrual cycle, cytokines and chemokines are abundant in the endometrium, which recruits leukocytes and affects their division and activation. By regulating the composition and function of local uterine leukocytes, cytokines and chemokines can further enhance and maintain local uterine inflammation, leading to tissue damage and indirect involvement in PD.

### 2.5. Changes of Leukocytes in PD

Menstruation represents a highly regulated inflammatory process, manifested as substantial leukocytes before the occurrence of menstruation [44]. The major leukocyte subsets, like uterine NEUT, natural killer (NK) cells, mast cells (MCs), EOS, and macrophages, constitute up to 40% of the total cells in the premenstrual endometrium [69]. There is also growing evidence that leukocytes are closely associated with PD.

#### 2.5.1. NEUT

In the peripheral blood of patients with PD, inflammatory metabolites of NEUT are increased [58, 70]. Serum neutrophil-to-lymphocyte ratio is significantly higher in adolescents with PD and premenstrual syndrome (PMS) [71]. In this sense, NSAIDs can bring pain-relief effects via modulating oxidative stress and ionized calcium (Ca^2+^) levels of NEUT in patients with PD through voltage-gated calcium channels (VGCCs) and transient receptor potential (TRP) cation ones [72].

#### 2.5.2. EOS

In addition to the classic inflammatory response, allergic inflammation dominated by EOS is also of utmost importance and usually not recognized in PD pathophysiology. Recently, the role of EOS in the reproductive system has attracted much attention. Studies have further found that EOS is present in the endometrium before and during menstruation [73, 74]. Besides, EOS can regulate local immune and inflammatory responses and play a key role in PD induction and development. The elevated levels of eotaxin can also cause EOS migration to the uterus, promoting inflammatory edema and congestion in this organ [75]. Eosinophil cationic protein (ECP), major basic protein (MBP), neurotoxin, and eosinophil peroxidase released by activated EOS can also cause the release of reactive oxygen species (ROS) and cytotoxic molecules, promoting inflammation and inducing endothelial damage to activated platelets, which lead to vasoconstriction and blood clotting and ultimately reduced blood flow and aggravated PD. ECP can further promote MC release histamine and exacerbate pain [76]. Further explorations into the relationship between EOS and menstruation can thus help better understand the pathogenesis of PD.

#### 2.5.3. MCs

Human MCs are derived from CD34^+^ and CD117^+^ pluripotent hematopoietic stem cells in the bone marrow. MC progenitor cells can be also transformed from protective immune cells to effective proinflammatory ones, thus participating in the inflammatory process of different tissues. They are additionally involved in the induction of acute inflammation and tissue repair during chronic inflammation. In the female reproductive system, MCs are mainly distributed in the myometrium and endometrium layers [77], which are closely related to uterine smooth muscles, fibroblasts, and collagens. The mediators released by MCs can effectively stimulate uterine smooth muscle contractions. Studies have accordingly shown that MC activation plays a critical role in the control of full-term and premature delivery [78, 79] and is essential in the progression of inflammatory bowel disease (IBD) [80], rheumatoid arthritis (RA) [81], and cutaneous vasculitis [82].

Therefore, inflammatory factors are directly linked with PD occurrence and development. In addition to classic pain-causing substances (such as PGs and LTs), the inflammatory response contributes to PD, mainly caused by cytokines (i.e., IL-6 and TNF*α*), chemokines (viz. Monocyte chemoattractant protein-1 [MCP-1] and eotaxin), and leukocytes (including, NEUT, EOS, and MC), which need further examinations.

## 3. Acupuncture for Inflammatory Visceral Pain

Acupuncture is widely used in treating visceral pain, in which enhancing anti-inflammatory effect is assumed as one of the important mechanisms. Considering a large number of acupuncture forms for visceral pain, the use of this alternative therapy for inflammatory visceral pain is delineated here.

### 3.1. Clinical Evidence

According to a randomized controlled trial (RCT), comparing EA and medical treatment in 54 patients with chronic prostatitis/chronic pelvic pain syndrome (CP/CPPS) of category IIIB, EA (4 mA, 99 Hz) had significantly increased the scores of pain reduction, quality of life, and total Chronic Prostatitis Symptom Index (CPSI) compared with medical treatment [83]. These results were consistent with the reports that 47 patients with CPPS had been treated with EA (continuous wave, 3 Hz). After treatment, the levels of IL-8, IL-10, and TNF*α* in prostatic fluid had decreased, and the CPSI score had dropped. Acupuncture could thus have a significant effect on the treatment of CPPS, which could achieve anti-inflammatory and analgesic effects by reducing the levels of inflammatory factors [84]. Similarly, another RCT on 144 patients had demonstrated that EA (alternating wave, 2/15 Hz) had alleviated pain symptoms and improved quality of life concerning chronic pelvic pain in patients with the sequelae of pelvic inflammatory disease [85]. Acupuncture can further modulate the immune function in cases with irritable bowel syndrome (IBS), which is majorly manifested by downregulating the level of serum inflammatory factor IL-18, IL-23, and TNF*α* and reducing the number of MCs in the colon, to improve pain, intestinal gas, bloating, and stool consistency composite score [86]. In a pilot study, TEAS at Zusanli (ST36) and Neiguan (PC6) acupoints had similarly reduced rectal sensitivity in patients with IBS as manifested by increasing the threshold of rectal sensation of gas, desire to defecate, and pain [87]. One other pilot study had further suggested that acupuncture could effectively decrease subjective pain in pediatric patients with acute appendicitis and downregulate the white blood cell count. Therefore, this alternative medicine could be exploited as an effective nonpharmacological intervention for the treatment of acute appendicitis pain in children [88].

### 3.2. Animal Studies

Acupuncture has been shown to have several beneficial effects in animals with intestinal disorders. A recent study had accordingly revealed that EA (10 Hz, 1 mA, plus width 0.4 ms) at ST36 could reduce the production of inflammatory cytokines by activating *α*7nAChR-mediated JAK2/STAT3 signaling pathway in macrophages, thereby suppressing gastrointestinal inflammation and promoting its motility [17], which demonstrated the anti-inflammatory and analgesic effects of EA through vagus nerve from the point of view of the complete nerve circuit. Another recent study showed that EA at ST36 with low intensity (0.5 mA) can activate sensory neurons expressing PROKR2+, thus driving the vagal-adrenal axis to play a systemic anti-inflammatory effect, and pointed out that the anti-inflammatory effect of acupuncture was related to the intensity of stimulation and the depth of acupuncture [89]. EA at ST36 also has an ameliorating effect within inflammatory environments by decreasing inducible nitric oxide synthase (iNOS) expression, increasing serum IL-10 level by square wave pulses with 100 Hz, 1 mA, [90], and downregulating serum TNF*α* and IL1-*β* and colonic TNF*α* messenger ribonucleic acid (mRNA) expression by the intermittent pulse with 2 Hz frequency and 4 mA intensity [91]. EA at Tianshu (ST25), Zhongwan (CV12), and Shangjuxu (ST37) (alternating wave, 6/30 Hz) can further augment serum IL-4 content and moderate colonic NF-*κ*B p65 protein expression [92], and EA (15/25 Hz, 0.1–0.2 mA, 2–4 V) at ST36, Guanyuan (CV4) could modulate the balance between the splenic regulatory T cells and T-helper 17 lymphocytes in ulcerative colitis [93]. Manual acupuncture at neurogenic spots with slight modification could alleviate the body weight changes and diarrhea scores and normalize the increased level of myeloperoxidase activity, TNF*α*, and IL-1*β* in the colitis rats [94].

Briefly, both clinical evidence and animal studies have proved the efficacy and reliability of acupuncture in the treatment of inflammatory visceral pain, such as CCPS, IBS, ulcerative colitis, and acute appendicitis. The effects of this form of alternative medicine on the regulation of various inflammatory factors are thus involved in its pain-relief mechanism.

## 4. Anti-Inflammatory Mechanisms of Acupuncture-Related Therapies for PD

**Figure 1:**
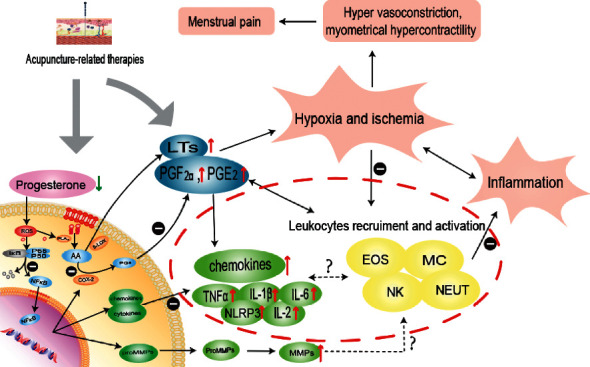
Possible anti-inflammatory effects of acupuncture-related therapies contributing to analgesia in menstrual pain. Progesterone withdrawal leads to the increased production of prostaglandins (PGs), leukotrienes (LTs), cytokines, chemokines, and matrix metalloproteinases (MMPs). These inflammatory factors promote the recruitment and activation of leukocytes, further increasing the inflammation and uterine hypoxia and ischemia, ultimately aggravating menstrual pain, which are considered as a vicious cycle. Acupuncture could reduce the release of PGs and downstream inflammatory cytokines by regulating nuclear factor-*κ*B (NF-*κ*B) signaling pathway, further relieving the inflammatory environment of uterus and uterine ischemia and hypoxia to alleviate menstrual pain. We suggest that further complexity is added to acupuncture-induced analgesia via interactions between chemokines and leukocytes. Symbols “↑” represent rising trend during menstrual pain. Symbols “—” represent inhibition of acupuncture. Abbreviations are listed at the end of the article.

As discussed earlier, acupuncture can bring a good effect on inflammatory visceral pain. PD, as a common visceral pain condition, is also closely related to inflammatory factors. Previous studies have mostly focused on the effects of acupuncture on PGs and analgesics. In contrast, there has been little research on the treatment of PD from an anti-inflammatory perspective, to the best of the authors' knowledge. Therefore, it is necessary to explore how acupuncture affects inflammatory factors in the treatment of PD and reflect on the influence of inflammatory factors on this condition. The study findings accordingly revealed that acupuncture-related therapies have shown satisfactory effectiveness in treating dysmenorrhea by enhancing the anti-inflammatory effects. The possible inflammatory mechanisms of menstrual pain and acupuncture-related therapies can thus alleviate menstrual cramps by mediating relevant inflammatory pathways, as illustrated in Figure 1.

### 4.1. Acupuncture

As mentioned, cytokines, chemokines, and inflammatory cells play a critical role in dysmenorrhea pathophysiology. Acupuncture has been further shown to exert a satisfactory effect on menstrual cramps; however, the mechanism of this form of alternative medicine for PD has not been fully explained. Some experimental studies have further reported that acupuncture can reduce the levels of inflammatory factors and immune cells in the rat model of dysmenorrhea.

In this respect, Zhao et al. have found that EA (dense wave, 50 Hz) at CV4 and Sanyinjiao (SP6) acupoints had significantly reduced the writhing response and the contents of IL-2, 5-HT, and substance P in the serum of rats with dysmenorrhea [95]. These results were consistent with the reports by Luo et al., in which EA (dense wave, 50 Hz) at CV4 and SP6 had mitigated the levels of TNF*α* and IL-1 in the serum of rats with PD, relieved uterine contractions, and alleviated pain [96], denoting that EA could alleviate menstrual pain and the mechanism was related to the reduction of peripheral inflammatory factors.

In rats with dysmenorrhea, the CD3 and CD4 levels and the ratio had further decreased, and the immune organs, namely, the thymus gland and the spleen, had shown obvious pathological changes. Li et al. have also reported that acupuncture at Zhibian (BL54) acupoint could significantly minimize the writhing response and consequently increase the levels of T lymphocyte subsets of CD3, CD4, CD4, and CD8 in the peripheral blood [97]. Similarly, Ju et al. have established that EA (dense wave, 50 Hz) could not only improve the CD3 and CD4 levels but also develop pathological changes in the thymus gland and the spleen [98]. These studies have indicated that EA could relieve pain by enhancing the immune function in rats with PD.

### 4.2. Moxibustion

Herbal cake-partitioned moxibustion (HM) is characterized by the combination of moxibustion with traditional Chinese medicine (TCM) and is being applied more widely than conventional moxibustion in clinical applications, especially in China [99].

HM can significantly reduce the writhing times, upregulate plasma beta-endorphin (*β*-EP) as well as uterus PGE_2_ content and splenic NK cell activity, and even downregulate uterus PGF_2*α*_ levels in rats with dysmenorrhea [100]. MCs also have a wide range of relationships and functions in the neuroendocrine-immune network. HM can thus upregulate the number and degranulation rate of MCs in the Shenque (CV8) acupoint [101] and downregulate the expression of MCs in the uterus [102]. Another research had further shown that HM could achieve therapeutic effects by downregulating the high expression of upstream transient receptor potential vanilloid (TRPV) in the uterine MCs of rats with dysmenorrhea. Moreover, after pretreatment with MC membrane stabilizer (that is, sodium cromoglycate), the analgesic effect of HM could be weakened by the inhibitory effect of sodium cromoglycate on the function of MCs in the CV8 acupoint, suggesting the specific role of MCs in the analgesic effect of HM [103]. In addition, moxibustion could downregulate NF-*κ*B expression and inhibit the release of TNF*α* and IL-2 in rats with dysmenorrhea [104].

### 4.3. ACE Treatment

ACE is a form of TCM external therapy with some absorbable catgut suture implants into the acupoints. In addition, it is characterized by the advantages of easier operation and durable stimulation, compared with acupuncture [105]. ACE can also have a significant effect on dysmenorrhea, which may be related to its impact on inflammatory factors. Recently, studies have shown that ACE can significantly improve symptoms and pathological damage in rats with PD, downregulate NLR family pyrin domain containing 3 (NLRP3), Caspase-1, IL-1*β*, and IL-18 protein expression in uterine tissues [106]; upregulate plasma *ß*-EP, uterus PGE_2_ content, and splenic NK cell activity; and even decrease PGF_2*α*_ content [100, 107] and downregulate COX-2 and NF-*κ*B p65 protein expression in uterine tissues [108].

As a whole, acupuncture and its related therapies have partly shown that anti-inflammatory effects contribute to analgesic mechanisms on PD. It is worthwhile to study further to enrich the multitarget and multilink mechanism of clinical treatment of this debilitating condition.

## 5. Recommendations for Future Researches

With the understanding of the relationship between inflammation and dysmenorrhea deepens, the treatment of this common gynecological disorder from an anti-inflammatory perspective becomes promising. Given this, it is speculated that enhancing anti-inflammatory effects is one of the important mechanisms of acupuncture as an alternative medicine for PD.

Based on the recent acupuncture experimental studies summarized above, we make some recommendations for future researches as follows:

1. Targeting anti-inflammatory effect of acupuncture in the treatment of PD from the perspective of chemokine-mediated inflammatory response will be a new direction to understand the mechanism of acupuncture for PD. At present, the anti-inflammatory effect of acupuncture on PD is mostly discussed from the aspects of inflammatory cells and cytokines (e.g., TNF*α*, IL-1, NF-*κ*B, NK cell activity, and MC). For further studies, it is interesting to explore the effects of acupuncture on chemokines since the latest evidence has shown that the serum levels of eotaxin have an increasing trend in patients with PD [68].
(2) Optimizing acupuncture intervention methods according to classic acupuncture theory is crucial in future mechanism studies of PD. Clinical and experimental studies have indicated that alterations in needling techniques may influence the therapeutic outcomes of acupuncture [109–111]. Dysmenorrhea with the congealing-cold syndrome is the most common pattern in TCM [112]. Previous clinical and animal studies have accordingly provided evidence that transverse needling on SP6 has outperformed in relieving menstrual pain with the congealing-cold syndrome via different pathways [113–115], supporting a classic acupuncture theory of “transverse needling benefits for reinforcing yang organs of the human body”. Therefore, it is recommended to conduct future acupuncture studies in line with the notion of emphasizing stimulation modes rather than acupoint selection alone. Meanwhile, it is also suggested to probe the anti-inflammatory mechanism of acupuncture with different needling methods for PD. Such studies are of great significance to increase the awareness of the benefits of acupuncture to optimize its clinical modalities for this disorder.
(3) The effects and mechanism of more convenient self-healing approaches for PD should be investigated. Although there are numerous acupuncture-like methods to reduce menstrual cramps, a more convenient procedure for patients is urgently needed because PD interferes with the activities of daily living and work of young women. TEAS/TENS are also among the noninvasive treatment options to stimulate acupoints or sites on the surface of the body, which have the advantages of being safe, easy to use, and digitally operated. Studies have further shown that TENS/TEAS can treat many types of pain [116], including inflammatory pain [117] with satisfactory results [118]. A clinical study had also revealed that TEAS could decrease the levels of CXCL8, IL-1, IL-6, TNF*α*, and CCL2 to relieve inflammatory responses and decrease the injuries caused by lower limb ischemia-reperfusion [119]. Another RCT had further established that TEAS could facilitate postoperative rehabilitation and even reduce stress response, and surgical inflammation in elderly patients undergoing knee surgery [120]. An animal study had correspondingly focused on the effect of TENS on the process of wound healing from the perspective of proinflammatory cytokine expression and had found that TENS had significantly mitigated the immunoreaction of TNF*α*, IL-1*β*, and IL-6 in the dermis compared with other treatments, indicating that TENS had shortened the healing process by inhibiting the inflammatory responses [121]. Meanwhile, studies had verified the effectiveness of TENS in reducing menstrual pain and improving the quality of life in patients with dysmenorrhea [122–124]. Compared with treatment methods such as acupuncture, TEAS has also eased restrictions on in-hospital treatment. Patients can even receive treatment at home and in the workplace. Certainly, the anti-inflammatory mechanism of TEAS/TENS with proper stimulation parameters in treating PD is worthy of further study.

## 6. Conclusion

Acupuncture and its related therapies achieve satisfied analgesic effects on PD via the downregulation of a wide variety of inflammatory cells and cytokines (e.g., TNF*α*, IL-1, IL-2, IL-18, COX-2, NF-*κ*B, NK cell activity, and MCs). The anti-inflammatory effects of acupuncture may further contribute to its analgesia in the treatment of PD, so there is a need to carry out more researches to confirm it. For further studies, it is interesting to explore the effects of acupuncture on chemokine-mediated inflammation with optimized acupuncture intervention methods according to classic acupuncture theory. Additionally, the effects and mechanism of more convenient self-healing approaches such as TENS/TEAS for PD should be investigated.

## Abbreviations

PD: Primary dysmenorrhea
NSAIDs: Nonsteroidal anti-inflammatory drugs
EA: Electroacupuncture
ACE: Acupoint catgut embedding
TEAS: Electrical acupoint stimulation
TENS: Transcutaneous electrical nerve stimulation
PLA2: Phospholipase A2
AA: Arachidonic acid
PGs: Prostaglandins
LTs: Leukotrienes
COX: Cyclooxygenase
LOX: Lipoxygenase
IL: Interleukin
TNF*α*: Tumor necrosis factors *α*
NF-*κ*B: Nuclear factor-*κ*B
MMPs: Matrix metalloproteinases
EOS: Eosinophil
NK: Natural killer cells
MC: Mast cells
NEUT: Neutrophils
PMS: Premenstrual syndrome
ECP: Eosinophil cationic protein
MBP: Major basic protein
ROS: Reactive oxygen species
RCT: Randomized controlled trial
CP/CPPS: Chronic prostatitis/chronic pelvic pain syndrome
CPSI: Chronic Prostatitis Symptom Index
IBS: Irritable bowel syndrome
HM: Herbal cake-partitioned moxibustion
TCM: Traditional Chinese medicine
iNOS: Inducible nitric oxide synthase
mRNA: Messenger ribonucleic acid
β-EP: Beta-endorphin
TRPV: Transient receptor potential vanilloid
NLRP3: NLR family pyrin domain containing 3.
